# Spatiotemporal impact of non-pharmaceutical interventions against COVID-19 on the incidence of infectious diarrhea in Xi'an, China

**DOI:** 10.3389/fpubh.2022.1011592

**Published:** 2022-11-28

**Authors:** Hui Zhang, Li Shen, Minghao Sun, Chenxi Zhao, Qian Li, Zurong Yang, Jifeng Liu, Kun Liu, Bo Xiao

**Affiliations:** ^1^Department of Prevention of Infectious Diseases, Xi'an Center for Disease Control and Prevention, Xi'an, Shaanxi, China; ^2^School of Remote Sensing and Information Engineering, Wuhan University, Wuhan, China; ^3^Department of Epidemiology, Ministry of Education Key Lab of Hazard Assessment and Control in Special Operational Environment, School of Public Health, Air Force Medical University, Xi'an, China; ^4^Department of Plastic Surgery, Xijing Hospital, Air Force Medical University, Xi'an, China

**Keywords:** infectious diarrhea, COVID-19, non-pharmaceutical interventions, short-term impact, long-term impact, Bayesian structural time series model

## Abstract

**Background:**

Non-pharmaceutical interventions (NPIs) against COVID-19 may prevent the spread of other infectious diseases. Our purpose was to assess the effects of NPIs against COVID-19 on infectious diarrhea in Xi'an, China.

**Methods:**

Based on the surveillance data of infectious diarrhea, and the different periods of emergence responses for COVID-19 in Xi'an from 2011 to 2021, we applied Bayesian structural time series model and interrupted time series model to evaluate the effects of NPIs against COVID-19 on the epidemiological characteristics and the causative pathogens of infectious diarrhea.

**Findings:**

A total of 102,051 cases of infectious diarrhea were reported in Xi'an from 2011 to 2021. The Bayesian structural time series model results demonstrated that the cases of infectious diarrhea during the emergency response period was 40.38% lower than predicted, corresponding to 3,211 fewer cases, during the COVID-19 epidemic period of 2020–2021. The reduction exhibited significant variations in the demography, temporal and geographical distribution. The decline in incidence was especially evident in children under 5-years-old, with decreases of 34.09% in 2020 and 33.99% in 2021, relative to the 2017–2019 average. Meanwhile, the incidence decreased more significantly in industrial areas.

**Interpretation:**

NPIs against COVID-19 were associated with short- and long-term reductions in the incidence of infectious diarrhea, and this effect exhibited significant variations in epidemiological characteristics.

## Introduction

The COVID-19 pandemic continues to be a global public health problem, and there were more than 575.88 million confirmed cases and 6.39 million deaths worldwide as of 2 August 2022 (1). The control of the pandemic has been difficult because of the emergence of novel variants of SARS-CoV-2 (Alpha, Beta, Gamma, Delta, and Omicron), some of which can escape vaccination, and the lack of specific and highly effective treatments (2–5). Thus, despite the availability of vaccines and drugs, non-pharmaceutical interventions (NPIs) are also important for controlling the spread of COVID-19 in China and elsewhere (6). China first implemented NPIs at a nationwide scale during the early stages of the pandemic (7), and has maintained a “dynamic COVID-zero” strategy. The goal of this strategy is to prevent the importation of new cases from outside China and the rebound of cases within China. Thus, when new local cases of COVID-19 emerge, comprehensive prevention and control measures are implemented, referred to as the “find one, extinguish one” approach. This strategy aims to rapidly block transmission and terminate each local epidemic, with the goal of providing maximum effectiveness at minimum cost (8). NPIs are an important part of the comprehensive prevention and control measures used to prevent COVID-19, and these include social mobilization, regional lockdowns, traffic control, cancellation of large-scale activities, closing of schools and workplaces, and home quarantine (9). Other measures include increasing the distance between uninfected and infected individuals; strengthening the management of case isolation and close contact tracing; increasing the screening for fever and use of nucleic acid testing; scanning of QR codes to monitor individuals who are outside; and increasing personal protective measures, such as wearing masks and hand hygiene (9). It is likely that the many NPIs implemented to control COVID-19 could also reduce the transmission of other infectious diseases, even those that have different routes of transmission, such as gastrointestinal transmission, sexual transmission, and vector transmission (10). It is therefore necessary to understand the impacts of NPIs on the transmission of other infectious diseases because it is important to reduce the prevalence of common infectious diseases during the post-pandemic period.

Infectious diarrhea is characterized by abdominal pain and inflammation, acute diarrhea, and vomiting due to infection by bacteria, viruses, fungi, protozoa, or other pathogens. The World Health Organization (WHO) reported that each year diarrheal diseases affect nearly 1.7 billion people and cause 525,000 deaths in children who are younger than 5 years-old (11). Even though many cases of infectious diarrhea are preventable and treatable, it remains a significant public health problem in China and elsewhere. In particular, infectious diarrhea can cause malnutrition and growth retardation in children, and can also prevent adults from working (11). China has a high incidence of infectious diarrhea, and it has been among the top three notifiable infectious diseases during recent years (12). In 2021, there were 1.33 million cases nationwide, corresponding to an incidence of 94.33/100,000 (13). Most previous studies that examined the impact of NPIs on infectious diseases have focused on respiratory infections, and very few have examined their effects on intestinal infections (12, 14, 15).

In this study, we examined the short- and long-term impacts of NPIs that were designed to control COVID-19 on the epidemiology of infectious diarrhea and the prevalence of causative pathogens in the city of Xi'an, China. We examined data on infectious diarrheal diseases in Xi'an before, during, and after the first COVID-19 epidemic in China, and used a Bayesian structural time series (BSTS) model to evaluate the effect of NPIs on the geographical and demographic heterogeneities of infectious diarrhea. Our results provide a reference for the implementation of future measures that could help prevent and control infectious diarrhea in Xi'an and other large cities.

## Materials and methods

### Study area

Xi'an, the capital of Shaanxi Province in Western China, has an area of 10,752 square kilometers, 13 counties, and a population of 12.81 million in 2021. Infectious diarrhea has been among the top three infectious diseases in Xi'an during the past 10 years. Xi'an is also adjacent to Hubei province, whose capital city Wuhan was the epicenter of the initial COVID-19 outbreak (16). Thus, Xi'an was seriously affected by COVID-19 during the early days of the epidemic, from January 25 to March 31, 2020. Starting from March 23, 2020, Xi'an International Airport was one of 12 airports to which flights destined for Beijing were diverted (17). Since then, Xi'an has faced the threats of local and imported COVID-19 outbreaks, and this led the government to implement NPIs as strict prevention and control measures. This motivated our retrospective study of the impact of these NPIs on infectious diarrhea during the 2 previous years in the city.

### Data collection

#### Infectious diarrhea

Surveillance data of infectious diarrhea from Xi'an Municipal Center for Disease Control and Prevention were examined for the period of January 1, 2011 to December 31, 2021. For each case, gender, age, current address, occupation, reporting institution, date of onset, and date of etiological test result were collected. The weekly incidences in different age groups were analyzed in each of the 13 counties of Xi'an. These county-level population data were from the Xi'an Statistics Yearbook. Statistical methods were used to analyze the different patients and causative pathogens from 2011 to 2021. For each case, the sex, age group (<5 years, 5–20 years, >20 years), and county of residence were analyzed. Weather data of Xi'an from 2011 to 2021 were from the China Meteorological Data Sharing Service System (http://data.cma.cn/). The weather data included temperature, relative humidity, precipitation, evaporation, atmospheric pressure, and duration of sunlight. The geographic boundaries of Xi'an were from the National Catalogue Service for Geographic Information of China (https://www.webmap.cn) and base maps were from the ArcGIS Online platform of ESRI (https://server.arcgisonline.com/).

#### NPIs against COVID-19

The specific public health emergency response in China has four levels (18). Level 1 has the strictest public health interventions. Xi'an reported its first confirmed local case of COVID-19 on January 23, 2020, and the government implemented a city-wide Level 1 emergency response 2 days later (19). This included implementation of a series of the strictest NPIs. On February 28, 2020, the local government downgraded the public health emergency response to Level 3, but the strict NPIs remained in place until the end of March (20). People began to return to their normal lives in April, but were still required to wear masks, undergo nucleic acid testing, scanning of QR codes, and mandatory quarantine if necessary. While continuing to implement COVID-19 prevention and control policies formulated by the city government, all kindergartens, schools, and universities resumed classes from June 9 to July 14 (spring semester, summer). Notably, summer has a low incidence of respiratory infectious diseases in Xi'an city. The fall semester began on September 1 of 2020, as usual. Then, schools and kindergartens gradually entered the season of high incidence of respiratory infectious diseases. At this time the NPIs had a greater impact on population behavior, especially for school students and kindergarteners, although the NPIs were more relaxed than during the previous half-year. The last known local case of COVID-19 in Xi'an was in February in 2020, and all newly reported cases were imported from outside the city until the identification of a local case on January 28, 2021. At that time, the Xi'an government immediately tightened their NPIs and advised students and others to avoid non-essential travel during the Winter Holiday and Spring Festival Holiday. Since then, several sporadic cases and small outbreaks of COVID-19 continued to occur in Xi'an city throughout the year. According to the number of cases, the city government implemented a series of targeted NPIs to achieve prevention and control. Based on the impacts of the intensity, duration, and frequency of the local interventions against COVID-19 on the population behavior in Xi 'an, especially students and kindergarteners, and followed by the epidemic characteristics of the respiratory infectious diseases, the COVID-19 epidemic in Xi'an was divided into four time periods: pre-COVID19 Period (1st week of 2011 to 4th week of 2020); Period 1 (P1, 5th week to 42nd week of 2020); Period 2 (P2, 43rd week of 2020 to 13th week of 2021); and Period 3 (P3, 14th week to 52nd week of 2021).

### Data analysis

The Xi'an government strengthened its reporting of infectious diseases since 2017 due to an assessment of the National Basic Public Health Service project and the allocation of additional resources. Based on this change, the pre-COVID19 period was subdivided into two sub-periods:1st week of 2011 to 52nd week of 2016, and 1st week of 2017 to 4th week of 2020. The incidences of infectious diarrhea were then compared for the different time periods. A BSTS model was used to predict the incidence of infectious diarrhea in Xi'an and to calculate differences between predicted and reported incidences. The same procedure was applied in separate analyses of each of the 13 counties. Weather data were added to the BSTS model as covariates to improve the forecast results.

The time series data before and after implementation of the NPIs were analyzed using segmented regression. An interrupted time series analysis (ITSA) component was used in the BSTS model after accounting for seasonality and autocorrelation. ITSA is a valuable tool that provides robust results regarding the effectiveness of different population-level health interventions at different times by comparing the incidence of a disease before and after the intervention (21, 22). To assess the impacts of NPIs on infectious diarrhea at different stages, the incidence of infectious diarrhea was determined for P1, P2, and P3. Three indicator variables (X1, X2, and X3) represented the implementation of interventions during the three periods, and were coded as 0 (before the intervention) or 1 (after the intervention). The changes in incidence after each intervention were measured using the regression coefficients of the three indicator variables (X1, X2, and X3), and changes in the slope of incidence were measured using the regression coefficients of the product of a continuous-time variable T and X1, X2, and X3. The inclusion probability reflects the likelihood of the change. Here Beta represents the corresponding regression coefficients.

All analyses are implemented in R, version 4.0.4, and the package of BSTS 0.9.7 was used to build the BSTS model. The thematic maps of infectious diarrhea incidence were made by ArcGIS 10.8 software.

### Ethical statement

In China, the collection of data from infectious diarrhea cases is part of routine public health surveillance, and such data collection is exempt from institutional review board assessment. Ethical approval for this study was not required in accordance with local legislation and national guidelines.

## Results

### Epidemiology of infectious diarrhea

**Figure 1:**
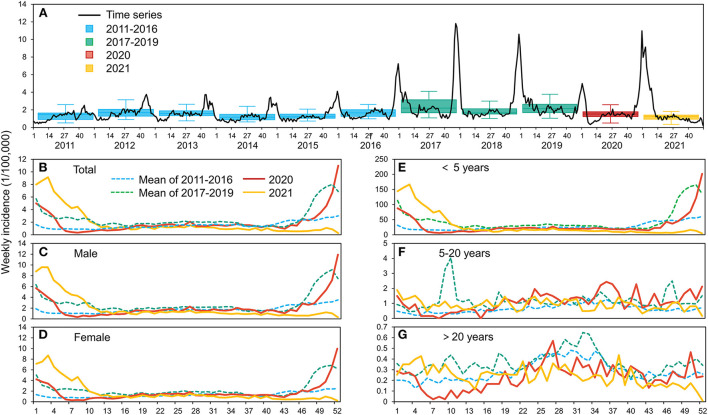
Weekly incidence of infectious diarrhea in Xi'an from 2011 to 2021 **(A)**, and average weekly incidences during four different time periods with stratification by sex **(B–D)** and age **(E–G)**.

From January 1, 2011 to December 31, 2021, there were 102,051 cases of infectious diarrhea were reported in Xi'an (Figure 1A). The winter peak was typically from the 44th week of one year to the 4th week of the following year, and cases reported during this 12-week period accounted for 40.20% of the total (41,023/102,051). The summer incidences were all very low. The average annual incidence from 2017 to 2019 (133.47/100,000) was higher than the average from 2011 to 2016 (78.87/100,000). After the onset of the COVID-19 epidemic, the annual incidences in 2020 (99.58/100,000) and 2021 (98.81/100,000) were significantly lower than the 2017 to 2019 average.

Separate analysis of males and females (Figures 1B–D) also showed high average annual incidences from 2017 to 2019 (males: 148.20/100,000; females: 117.78/100,000), and lower incidences during 2020 (males: 109.67/100,000, 26.00% decrease; females: 88.74/100,000, 24.66% decrease) and 2021 (males: 105.58/100,000, 28.76% decrease; females: 91.74/100,000, 24.66% decrease). Separate analysis of the three age groups (Figures 1E–G) indicated that children who were < 5 years-old accounted for 80.72% (82,375/102,051) of all cases, and the age group also experienced the greatest decline in incidence after the onset of COVID-19, with a decrease of 34.09% in 2020 (1,572.43/100,000) and 33.99% in 2021 (1,574.85/100,000) relative to the 2017–2019 average (2,385.79/100,000). After the COVID-19 outbreak, seasonal variations in the incidence of infectious diarrhea were similar in each group. At the beginning of 2020, the incidence was lower than the same period of previous years.

### Causative pathogens

**Figure 2:**
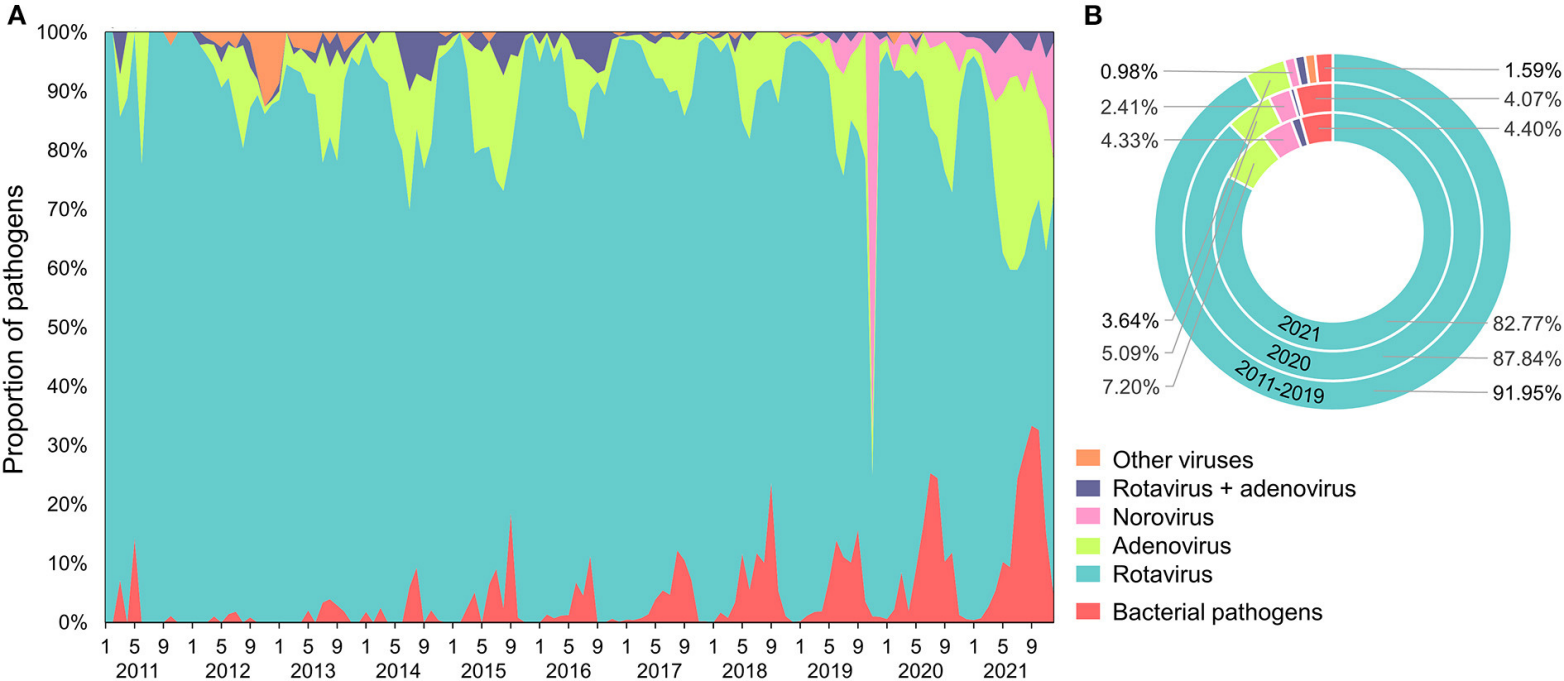
**(A)** Weekly and **(B)** yearly proportion of pathogens responsible for infectious diarrhea in Xi'an from 2011 to 2021.

During the entire study period, causative pathogens were identified in 20.78% of all cases (21,210/102,051), and rotaviruses accounted for 90.26% of this total (19,144/21,210; Figure 2). The other major pathogens were adenoviruses (4.28%; 908/21,210), bacteria (2.22%; 470/21,210), and the norovirus (1.59%; 338/21,210). Notably, the percentages of infections by bacteria (1.59%), adenoviruses (3.64%), and the norovirus (0.98%) were low from 2011 to 2019, and these percentages were all greater during 2020 (4.07, 5.09, and 2.41%) and 2021 (4.40, 7.20, and 4.33%). On the contrary, the percentage of infections by rotaviruses decreased over time (2011–2019: 91.95, 2020: 87.84, and 2021: 82.77%).

### Impact of NPIs

**Figure 3:**
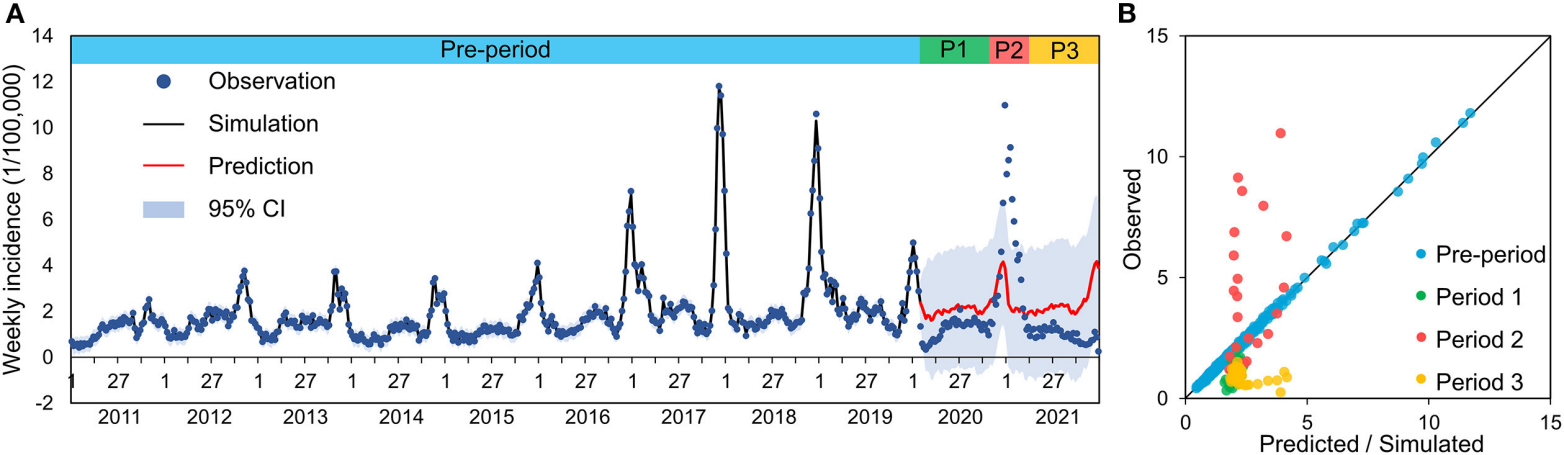
Observed incidence and predicted incidence (Bayesian structural time series model) of infectious diarrhea in Xi'an **(A)** and differences between predicted and observed values during different periods **(B)**.

During the initial phase of the COVID-19 epidemic (P1), the incidence of infectious diarrhea declined significantly, in parallel with implementation of the strictest NPIs (Figure 3). After relaxation of the NPIs (P2), the incidence quickly recovered to the previous level. The incidence dropped to the lowest level during the 8th week of 2020 (0.33/100,000). Thus, the incidence during P1 was 18.89% lower (46.42/100,000) than the comparable period from 2011 to 2019 (57.23/100,000). The BSTS results showed that the predicted incidences were higher than the reported incidences during P1 and P3, but the predicted incidence was much lower than the reported incidence during P2. We estimated that the number of cases during P1 (4,741) was 40.38% lower than predicted (7,952; 95% credible interval [CI]: −51.63, −21.23%). In other words, there were 3,211 fewer cases than predicted (95% CI: −5,060, −1,278) during P1.

For the period of 2020 to 2021, the 49th week of 2020 had the greatest cumulative reduction in the number of cases compared to the predicted number, with a total reduction of 3,657 cases (95% CI: −5,900, −1,453). The incidence (182.45/100,000) was 20.64% lower than expected (229.89/100,000), corresponding to 2,650 fewer cases (95% CI: −6,987, −2,159) during the COVID-19 epidemic from 2020 to 2021. Surprisingly, the reported incidence peak during the 52nd week of 2020 to the 3rd week of 2021was 2 weeks later than predicted (week 50 to week 51 of 2020), and the reported incidence peak (10.98/100,000) was much higher than predicted (4.15/100,000).

**Figure 4:**
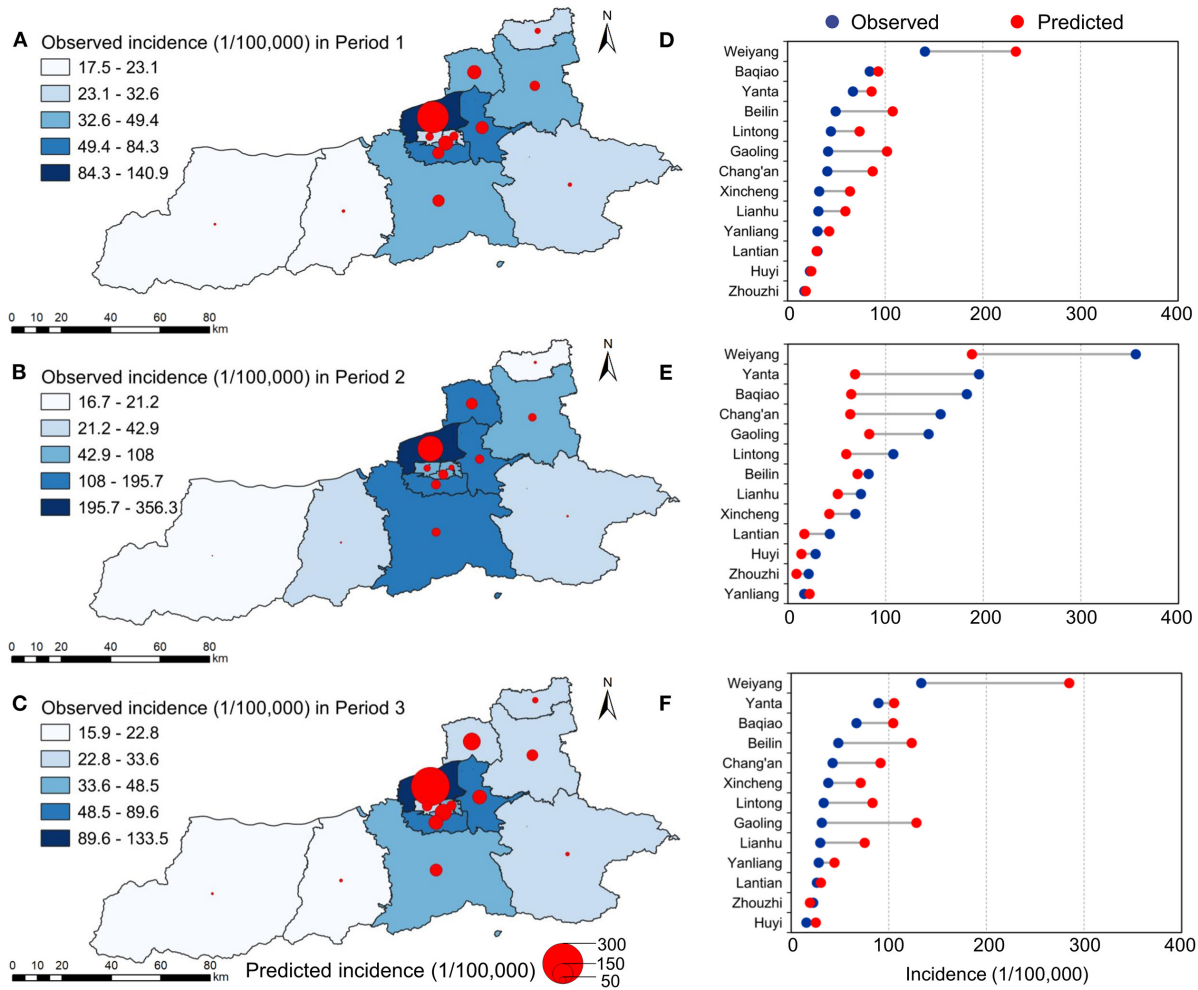
Observed incidence and predicted incidence maps of infectious diarrhea at county level in Xi'an during P1, P2, and P3 **(A–C)** and county-level differences between predicted and observed values during the different periods **(D–F)**.

We also used the BSTS with the same methods to predict the temporal variations in the incidence of infectious diarrhea in each county of Xi'an during the three post-epidemic periods (Table 1). The results showed that Weiyang (−96.13/100,000), Beilin (−60.77/100,000), and Gaoling (−53.50/100,000) had the greatest absolute differences between reported and predicted incidence during P1. Weiyang, Yanta, and Baqiao had the greatest absolute differences during P2, and Weiyang, Gaoling, and Beilin had the greatest absolute differences during P3. We found that higher predicted and reported incidences of infectious diarrhea were in Weiyang and surrounding counties (Beilin,Yanta, and Gaoling). The predicted incidences during P1 and P3 were generally higher than reported in most counties; however, the predicted incidences during P2 were generally lower than reported in most counties. The differences between the reported and predicted incidences were also greatest in counties that had higher total reported incidences (Figure 4). The model results showed that all counties had obvious seasonal changes, and all counties also had epidemic peaks during the winter of 2020 and 2021. These differences were mostly negative during P1 (range: −0.73–0.94, mean: −0.41) and P3 (range: −0.81–0.87, mean: −0.57), but the differences were mostly positive during P2 (range: −0.20–2.75, mean: 0.70). Among all 13 counties, the mean relative difference between reported and predicted incidence was greatest in Gaoling during P1, Yanta during P2, and Gaoling during P3.

**Table 1:** Cumulative differences between predicted (Bayesian structural time series model) and reported incidence of infectious diarrhea during the three post-COVID-19 time periods in Xi'an overall, and in 13 different counties of Xi'an.

**Region**	**Time periods**					
	**Period 1**		**Period 2**		**Period 3**	
	**Absolute difference**	**Relative difference**	**Absolute difference**	**Relative difference**	**Absolute difference**	**Relative difference**
Xi'an	−31.90 (−128.47,22.54)	−0.41 (−0.73,0.94)	40.65 (−24.20,72.71)	0.70 (−0.20,2.75)	−49.91 (−162.37,17.13)	−0.57 (−0.81,0.87)
Xincheng	−30.01 (−129.19,16.46)	−0.48 (−0.80,1.02)	28.20 (−35.84,44.90)	0.69 (−0.34,1.86)	−29.38 (−139.53,19.47)	−0.43 (−0.78,1.03)
Beilin	−60.77 (−166.06,42.38)	−0.55 (−0.77,6.02)	14.72 (−52.01,75.37)	0.22 (−0.39,10.26)	−68.23 (−178.71,39.77)	−0.58 (−0.79,4.56)
Lianhu	−26.87 (−134.22,16.44)	−0.46 (−0.81,1.07)	27.69 (−44.25,46.32)	0.59 (−0.37,1.63)	−36.80 (−157.45,14.31)	−0.55 (−0.84,0.90)
Baqiao	−9.18 (−111.33,78.38)	−0.10 (−0.57,13.14)	118.14 (51.48,144.67)	1.82 (0.39,3.76)	−33.72 (−151.59,53.82)	−0.33 (−0.69,4.03)
Weiyang	−96.13 (−437.72,69.83)	−0.41 (−0.76,0.98)	172.91 (−62.33,250.96)	0.94 (−0.15,2.38)	−141.53 (−559.14,75.81)	−0.51 (−0.81,1.31)
Yanta	−20.35 (−135.96,33.76)	−0.23 (−0.67,1.01)	131.04 (58.44,175.16)	2.03 (0.43,8.54)	−6.52 (−126.78,50.41)	−0.07 (−0.59,1.29)
Yanliang	−13.88 (−68.66,27.24)	−0.31 (−0.69,7.44)	−4.31 (−39.53,9.16)	−0.21 (−0.70,1.22)	−16.89 (−80.33,15.62)	−0.37 (−0.74,1.22)
Lintong	−28.37 (−140.65,23.75)	−0.39 (−0.76,1.14)	48.35 (−26.09,91.17)	0.81 (−0.19,5.42)	−42.10 (−171.17,18.54)	−0.56 (−0.84,1.23)
Chang'an	−40.96 (−170.66,18.68)	−0.50 (−0.81,0.84)	96.78 (10.45,150.34)	1.62 (0.07,24.68)	−43.76 (−192.69,21.25)	−0.51 (−0.82,0.99)
Gaoling	−53.50 (−296.82,20.86)	−0.56 (−0.88,1.00)	70.76 (−92.00,72.06)	0.96 (−0.39,1.00)	−73.84 (−352.88,15.83)	−0.70 (−0.92,1.00)
Huyi	−1.13 (−29.67,19.29)	−0.05 (−0.56,5.10)	15.44 (−2.92,23.19)	1.20 (−0.09,4.54)	−9.56 (−41.83,14.83)	−0.38 (−0.72,14.04)
Lantian	2.54 (−37.22,17.35)	0.09 (−0.55,1.28)	26.32 (1.17,21.44)	1.59 (0.03,1.00)	−2.75 (−46.63,15.65)	−0.09 (−0.64,1.43)
Zhouzhi	0.39 (−26.36,12.29)	0.02 (−0.60,2.38)	12.40 (−3.44,10.61)	1.41 (−0.14,1.00)	4.52 (−23.33,13.63)	0.25 (−0.51,1.48)

Absolute difference refers to cases/100,000 and relative difference refers to fractional change.

The impacts of NPIs (“interruptions”) on the incidence of infectious diarrhea were showed in Table 2. The results showed that the change in the level of coefficient X1 at the beginning of P1 had the greatest immediate effect in reducing the incidence of infectious diarrhea (inclusion probability: 0.9874), and its negative β2 coefficient confirmed the immediate drop of incidence after the implementation of the emergency NPIs. In other words, NPIs sharply reduced the incidence of infectious diarrhea during P1, at beginning in the 5th week of 2020. The lifting of the Level 1 emergency response decreased the intensity and variety of NPIs, and the association between NPIs and infectious diarrhea gradually decreased during P2 (inclusion probability: 0.6873) and P3 (inclusion probability: 0.1564). These results also showed that the slope change coefficient TX3 had the highest impact on infectious diarrhea incidence in the long-term trend (inclusion probability: 0.9976) and its β7 coefficient was negative. These results suggested that a trend of decreasing incidence of infectious diarrhea occurred after week 14 of 2021 compared to the previous times. The periodic changes of meteorological factors are generally not affected by NPIs, and these covariates included in the BSTS model could be better estimate the seasonal fluctuations of the disease. The meteorological covariates improved the fitting accuracy of the BSTS model, and its root mean square error (RMSE) decreased from 0.517 to 0.493.

**Table 2:** Regression coefficients of variables in the interrupted time series analysis.

	**Coefficients**		**Included coefficients**		**Inclusion probability**
	**Mean**	**SD**	**Mean**	**SD**	
β1(T)	0.0032	0.0004	0.0032	0.0004	1.0000
β2(X1)	−1.1349	0.2775	−1.1494	0.2476	0.9874
β3(X2)	1.6086	1.1494	2.3406	0.4567	0.6873
β4(X3)	0.7224	1.8221	4.6194	1.7974	0.1564
β5(TX1)	0.0003	0.0071	0.0131	0.0468	0.0215
β6(TX2)	0.0142	0.0223	0.0459	0.0125	0.3099
β7(TX3)	−0.0544	0.0282	−0.0546	0.0282	0.9976

SD, standard deviation; β, regression coefficient.

## Discussion

The Chinese government quickly implemented a series of strict NPIs soon after the onset of the COVID-19 outbreak in Wuhan, and these NPIs were very effective initially and remained in place for a long time (23, 24). The COVID-19 pandemic has had serious impacts on public health worldwide (16, 25). We therefore quantitatively assessed the short- and long-term effects of NPIs on infectious diarrhea in Xi'an. The results showed that during the COVID-19 epidemic, the incidence of infectious diarrhea experienced a more significant rebound to its previous seasonality, after a prominent decline due to the anti-COVID-19 NPIs. However, in the long term, the NPIs interrupted the upward trend in the incidence of infectious diarrhea, and there were lower annual incidences in 2020 and 2021 than the 2017–2019 average. At the same time, the NPIs led to major changes in the incidences of pathogens whose levels are usually relatively stable: the proportions of norovirus, adenovirus, and bacteria increased rapidly, and the proportion of rotavirus decreased. The impact of NPIs on the incidence of infectious diarrhea varied in different areas, in that industrial areas and areas with a dense permanent populations (especially students and kindergarteners) were more affected.

Before the COVID-19 pandemic, the average incidence of infectious diarrhea had a pronounced seasonal trend. There was also an increase over time, in that the incidence was higher from 2017 to 2019 than 2011–2016. This is consistent with the characteristics of a nationwide epidemic, as reported by Luo (26). After the first COVID-19 case was confirmed, the Xi'an government immediately adopted a series of very strict NPIs. The incidence of infectious diarrhea soon decreased significantly, and was at the lowest level in nearly 12 years by the eighth week of 2020. The time when the strictest NPIs were in place also had a greatly reduced peak of infectious diarrhea (winter of 2019 and 2020), with far fewer cases than predicted. Our ITSA results also confirmed that the very strict NPIs that were implemented during P1 led to an immediate and significant decrease in the incidence of infectious diarrhea.

Several factors may explain these observations. Firstly, during P1 people tended to move about less, they had increased awareness of the need for personal hygiene and healthy behaviors, and they paid more attention to hand hygiene and wearing masks (27). These changes could reduce the transmission of infectious diarrhea, which is mainly spread by the fecal-oral and respiratory routes (28–30). Secondly, the very strict NPIs reduced the demand for treatment of infectious diarrhea in patients who had mild symptoms, because they might have neglected treatment due to fear of exposure to COVID-19 (31). Thirdly, many hospitals reduced their outpatient visits and inpatients because medical personnel were deployed to respond to COVID-19.

Our results showed that the incidence of infectious diarrhea in Xi'an gradually increased when the city entered the stage when there were less intensive COVID-19 prevention and control measures, but the incidence did not exceed the previous level until the next seasonal peak in 2020 to 2021. We can suggest two reasons for this. Firstly, the strictest NPIs were implemented intermittently, and COVID-19 cases imported from abroad and sporadic local cases continued to occur. The NPIs likely had a long-term impact on people's behaviors, and this led to persistently low levels of infectious diarrhea. Secondly, medical resources became available for some patients who had infectious diarrhea with severe symptoms, especially because schools resumed classes on June 8, 2020.

It was somewhat unexpected that the incidence peak of infectious diarrhea was much higher than predicted during the winter of 2020 to 2021. This may be because of increases in the tourism, leisure, catering, and entertainment industries of Xi'an after the NPIs were relaxed (12). In addition, winter is also the season when the incidence of infectious diarrhea is the highest Xi'an (32). During P2 of the COVID-19 epidemic, the impact of NPIs persisted but gradually diminished as there were no indigenous cases for a long time, and this might explain why the winter peak of infectious diarrhea was 2 weeks later than predicted (16). The NPIs against COVID-19 dramatically increased public health awareness and hygiene practices, and people complied with these protocols by wearing masks, maintaining social distance, using health QR codes, washing their hands, and undergoing nucleic acid testing (33–35). These behaviors likely slowed the upward trend in the incidence of infectious diarrhea over the long term, so there was a lower annual incidence in 2020 and 2021 than the average from 2017 to 2019. This means that NPIs against COVID-19 provided sustainable benefits in reducing the incidence of infectious diarrhea. The results of our ITSA also confirmed that the intermittent NPIs that were implemented during P3 did not immediately change the incidence of infectious diarrhea, but changed their long-term trends.

There were only limited changes in the specific pathogens responsible for infectious diarrhea in Xi'an from 2011 to 2019, but there were major changes after the COVID-19 epidemic. The proportions of the norovirus, adenoviruses, and bacteria increased rapidly, and the proportion of rotaviruses decreased. We believe there were three reasons for this. Firstly, during the pandemic, schools remained open and classes mostly operated as normal as possible. Schools therefore may have facilitated the transmission of the norovirus and adenoviruses *via* touching of contaminated surfaces or close contact among students (36). Secondly, as a result of the NPIs against COVID-19, parents had fewer opportunities to socialize and likely urged their families to pay more attention to hygiene, so that parents and children were behaving similarly during the pandemic (21). These activities would likely reduce the transmission of rotaviruses to young children (37), and could therefore explain the large reduction in the incidence of infectious diarrhea in children younger than 5 years-old (38). Thirdly, an investigation by Wang et al. reported that NPIs significantly suppressed the spread of acute diarrhea caused by common enteroviruses throughout China, but this effect was not as strong for acute diarrhea caused by intestinal bacteria (39). This might be because people—especially children—who have bacterial diarrhea are more likely to experience fever and severe symptoms and are also more likely to seek medical care, even during the COVID-19 pandemic (40).

Our results indicated that NPIs had different effects on the incidence of infectious diarrhea in the 13 counties of Xi'an. The differences between reported and predicted incidences were greatest in counties with higher reported incidences. This might be because of differences in socio-economic structure, demographic characteristics, regional prevention and control policies, and population mobility among the counties. Differences in access to health services, the locations of schools, and population susceptibility can also contribute to spatial heterogeneity. For example, Gaoling county is an industrial area and provides jobs to many people in Xi'an. Thus, many residents of Beilin and Yanta counties pass through Weiyang to work in Gaoling. If they become sick, they might go to a hospital in Weiyang on weekdays, because this county has more medical institutions and better health services. This may explain the higher predicted and reported incidences of infectious diarrhea in Weiyang and surrounding counties.

During P1 and P3 of the COVID-19 epidemic, the incidences of infectious diarrhea in most counties decreased, and the mean relative differences were lower than the predicted, especially in Gaoling. Gaoling has the smallest resident population, but a large “floating population”. The local government implemented the most stringent NPIs, and this likely led to the greatest difference between the predicted and actual incidences of infectious diarrhea. During P2, a period with a seasonally high incidence of infectious diarrhea, the incidence in most counties increased and the mean relative difference was higher than predicted, especially in Yanta. Yanta has the largest number of students and children in kindergarten, and these individuals are more likely to seek treatment for mild infectious diarrhea.

This study has several limitations. Firstly, our study was observational and the relationships should be considered as correlations, not proof of causality. Secondly, the uncertainties and complexities created by the COVID-19 pandemic mean that additional direct evidence is needed to establish a link between individual behaviors with the risk of infection by enteric pathogens. However, it is difficult to quantify the impacts of NPIs of different intensities on overall population behavior. It is also difficult to obtain specific data on NPIs, such as the use of masks and disinfectants. Thirdly, rotaviruses were the predominant pathogen responsible for infectious diarrhea in Xi'an, and the only pathogen for which there is a vaccine in China (41). However, vaccination data is difficult to collect because parents pay for these vaccines at different medical institutions. The unavailability of these data therefore limited our analysis. Despite these limitations, the results of this study still suggested that NPIs reduced the incidence of infectious diarrhea in Xi'an. These results can thus serve as a reference for policy-makers whose aim is to reduce intestinal infectious diseases and improve public health in other large cities.

## Conclusions

Our study demonstrated that implementation of NPIs–especially the strictest NPIs–to control COVID-19 were associated with significantly reduced short-term and long-term incidences of infectious diarrhea and alterations in the spectrum of pathogens responsible for infectious diarrhea. In addition, the effects of these NPIs on infectious diarrhea differed according to time, geography, and patient age. Notably, we found that the greatest effects were in children younger than 5-years-old. These results indicated the need to strengthen and improve surveillance strategies for infectious diarrhea during the COVID-19 pandemic. Our findings have implications for the future development of public health interventions that seek to reduce the incidence of infectious diarrhea.

## Data availability statement

The raw data supporting the conclusions of this article will be made available by the authors, without undue reservation.

## Ethics statement

Ethical review and approval was not required for the study on human participants in accordance with the local legislation and institutional requirements. Written informed consent for participation was not required for this study in accordance with the national legislation and the institutional requirements.

## Author contributions

Conceptualization and writing—review and editing: BX and KL. Investigation, resources, and data curation: HZ, QL, and JL. Methodology, visualization, and formal analysis: HZ, LS, MS, and CZ. Writing—original draft: HZ, LS, and MS. All authors contributed to the article and approved the submitted version.

## Funding

This study was supported by the Natural Science Basic Research Project of Shaanxi Province, (Grant Numbers: 2020JQ-969 and 2020JM-329) and National Natural Science Foundation of China, (Grant Number: 81803289). The funders had no role in study design, data collection and analysis, decision to publish, or preparation of the manuscript.

## Conflict of interest

The authors declare that the research was conducted in the absence of any commercial or financial relationships that could be construed as a potential conflict of interest.

## Publisher's note

All claims expressed in this article are solely those of the authors and do not necessarily represent those of their affiliated organizations, or those of the publisher, the editors and the reviewers. Any product that may be evaluated in this article, or claim that may be made by its manufacturer, is not guaranteed or endorsed by the publisher.

## Abbreviations

NPIs: non-pharmaceutical interventions
WHO: World Health Organization
BSTS: Bayesian structural time series
ITSA: interrupted time series analysis
CI: credible interval.
